# The role of *BRCA1* and *BRCA2* mutations in prostate, pancreatic and stomach cancers

**DOI:** 10.1186/s13053-015-0038-x

**Published:** 2015-08-01

**Authors:** Helen Cavanagh, Katherine M.A. Rogers

**Affiliations:** School of Nursing and Midwifery, Queen’s University Belfast, 97 Lisburn Road, Belfast, BT9 7BL N. Ireland

**Keywords:** BRCA1, BRCA2, Non-breast/ovarian cancers, Prostate cancer, Pancreatic cancer, Stomach cancer

## Abstract

The association of germline mutations in the breast cancer susceptibility gene 1 (*BRCA1*) and the breast cancer susceptibility gene 2 (*BRCA2*) with the development of breast and ovarian cancers have been widely researched and recognised. It is known that these genes function at multiple sites in the body. Research has subsequently evolved into the connection of *BRCA1/2* with cancers at other sites within the body. This review examines the association of *BRCA1/2* germline gene mutations with prostate, pancreatic and stomach cancers. An extensive literature search revealed conflicting findings regarding the association of *BRCA1/2* gene mutations with these cancers. Most studies suggest that there is an association between *BRCA1/2* mutations and carcinoma of the prostate, pancreas and stomach, but some reports propose that such a correlation may be due to factors other than possessing a mutated *BRCA1/2* gene, and other associations may be revealed as further epidemiological information becomes available. The review concludes that as more knowledge arises about the mechanisms of *BRCA1/2* gene mutations, it should pave the way for future screening programmes to be applied effectively.

## Introduction

One of the major advancements in cancer research was the discovery of the *breast cancer susceptibility gene 1* (*BRCA1*) and the *breast cancer susceptibility gene 2* (*BRCA2*). Genetic abnormalities occur in all cancers therefore, as *BRCA1/2* pathways safeguard genetic content they are seen as critically important in research. *BRCA1/2* are tumour suppressor genes involved in pathways important for controlling DNA damage such as recognition, transcription regulation and double-strand break repair [1] and functions such as these are vital for all cell types to avoid developing mutations. The mechanisms by which *BRCA1/2* mutations lead to cancer of the breast and ovaries are not fully understood. One possible explanation for the particular targeting of the breasts and ovaries is that the epithelial tissue in these areas is particularly vulnerable to transformation [2]. It is known that *BRCA1/2* genes function at multiple sites throughout the body, not just in the breast and ovary. From this knowledge, research on *BRCA1/2* pathways and their impact in other cancers has evolved.

*BRCA1/2* genes are expressed in the cells of tissues around the body. These genes have multiple functional domains and interact with numerous proteins involved in many biological processes. Thus, as BRCA proteins function at a variety of sites, defects in DNA can occur in these different sites leading to the development of cancerous cells in tissues other than breast and ovarian [3]. *BRCA1* is reported to be involved in all phases of the cell cycle and regulates events during cell progression. The normal functioning *BRCA1* gene triggers cellular responses to DNA damage that can block cell proliferation and encourage apoptosis, subjecting cells to a high risk of malignant transformation [4]. The *BRCA2* gene is involved in repairing DNA. When mutations occur in *BRCA1/2* genes their normal function is disrupted, therefore, DNA damage can accumulate in a replicating cell resulting in the development of cancerous tissues in the area of the body where the mutated gene was situated [5].

Mutations in the *BRCA1/2* genes are classified as germline alterations since they are passed to subsequent generations through the male and female gametes (sperm and ovum respectively). The inheritable predisposition of the *BRCA1/2* genes for breast and ovarian cancers has been well established and widely researched. After the discovery of *BRCA1* on chromosome 17 [6] researchers believed that at least one other cancer gene was involved in hereditary breast and ovarian cancer hence the identification and location of the *BRCA2* gene on chromosome 13 [7] triggered widespread interest in genetic research and testing, leading to greater understanding of the mechanism underlying hereditary breast cancer.

Over the years research has highlighted the link between mutations in *BRCA1/2* genes and susceptibility to breast and ovarian cancer. It has been established that a woman’s risk of developing breast or ovarian cancer is greatly increased if she carries a mutated *BRCA1/2* gene [8]. It is estimated that approximately 60 % of women who carry a mutated *BRCA1/2* gene will develop breast cancer. Therefore, females with a mutated *BRCA1/2* gene are five times more likely to develop breast cancer than someone without a mutation [8, 9]. Nonetheless, *BRCA1/2* mutations are rare in the general population and cause approximately 2 % of all breast cancers diagnosed [10]. The connection with *BRCA1/2* gene mutations and female breast and ovarian cancers has been widely researched but not so for males. Until Edwards et al. [11] reported that men who carry a mutated *BRCA2* gene have an estimated 1 in 15 chance of developing breast cancer before they reached 70 years no such connections were made between males and *BRCA1/2*-related hereditary breast cancer. Breast cancer in men remains rare; latest statistics report approximately 350 cases of male breast cancer in the UK in 2011 compared to almost 50,000 cases in women [12].

This report reviews the most relevant available literature on the association of BRCA1/2 genes with prostate, pancreatic and stomach cancers.

## Review

This review focuses on the association of mutations in *BRCA1/2* with carcinoma of the prostate, pancreas and stomach and screening for *BRCA1/2* gene mutations. It is evident from the reviewed literature that the pancreas and prostate were the two most important sites for males who possess a *BRCA1/2* mutation.

### The impact of BRCA1/2 mutations for prostate cancer

When *BRCA1/2* mutations were discovered it was extensively reported that these mutations played a role in the development of breast and ovarian cancer. Since this advancement, considerable research has investigated whether *BRCA1/2* mutations bestow risk of prostate cancer. Familial aggregation of prostate cancer has been described and the germline mutation of *BRCA1/2* genes has been implicated in some research studies [13]. To date, many have reported on the association of both *BRCA1/2* mutations and some findings have indicated extensively lower rates of survival and more aggressive disease patterns [14, 15].

It is recognised that some prostate cancer diagnoses have a poor prognosis and this has been connected with hereditary factors [16]. The association between prostate cancer and *BRCA2* is consistent within research. Several studies indicate that male carriers of *BRCA2* are at an increased risk of developing prostate cancer [13, 17] while men with a *BRCA1* mutation are believed to have a slightly higher risk of developing prostate cancer than those who possess no *BRCA1/2* mutations. Early studies reported *BRCA1* mutation carriers to have a significant increased relative risk of developing prostate cancer [18]. Men who carry a mutated *BRCA2* gene are reported to be seven times more likely to develop prostate cancer than men who do not possess the mutation [19, 20]

*BRCA2* is a multisite cancer gene that not only affects women, but also increases a man’s risk of developing cancer [17]. There is a recognised association of breast cancer with prostate cancer within some families which has been highlighted within several epidemiological studies [21–23]. Agalliu et al. [24] found that risk of developing prostate cancer was particularly increased in participants who possessed a mutated *BRCA1* gene and had a first-degree relative with prostate, breast or ovarian cancer. Nonetheless, their findings showed that associations between founder mutations and prostate cancer were strong in men with no first-degree relative with breast or ovarian cancer and were unaffected by family history of prostate cancer [24]. Another study showed that two of 290 participants with prostate cancer possessed germline protein-truncating *BRCA2* mutations, giving an overall prevalence of 0.69 %. Only one of these participants reported a first-degree relative with prostate cancer and neither of the two participants reported a family history of breast or ovarian cancer [25].

Bermejo and Hemminki [26] demonstrated that families with bi-lateral breast cancer diagnosed before aged 50 years presented increased standard incidence ratios for prostate cancers. Edwards et al. [27] found that 18 % of participants in their study had a first-degree relative with prostate cancer. It was calculated that *BRCA2* mutations amount for approximately 6 % of the increased familial risk of prostate cancer. This suggests that while associations have been established, the overall contribution of *BRCA2* mutations to the familial aggregation of prostate cancer is small.

### Prostate cancer risk and age at diagnosis

It is frequently hypothesised that the risk of prostate cancer associated with mutations in *BRCA1/2* varies by age at diagnosis. Previously, an increased incidence was reported in men with *BRCA2* mutations and prostate cancer that were diagnosed before 65 years [20]. In some studies the risk of prostate cancer associated with *BRCA1/2* mutations is reported higher in men diagnosed at an older age [24]. Some studies also suggest that men with prostate cancer who harbour *BRCA1/2* mutations are believed to develop the disease at an early age. In the study by Agalliu et al. [24] participants were stratified into two groups: cases at age of diagnosis <65 and >65 years of age. Their findings demonstrated no associations of the founder mutations in the <65 year age group. They also showed a statistically significant result between the age group >65 years at prostate diagnosis and *BRCA1*. Edwards et al. [27] used the average of the estimated prevalence (0.12 and 0.07 %) of disease associated *BRCA2* mutations within the general UK population. It was reported that 50 % of the six prostate cancer patients carrying *BRCA2* mutations were diagnosed before aged 50 years. Similarly Agalliu et al. [25] reported the existence of aggressive prostate tumours developing at an early-onset, less than 55 years. This implies that screening may be required at a younger age among *BRCA1/2* mutation carriers.

Most often, the association between *BRCA1/2* mutations is assessed according to clinical features. In a study by Agalliu et al. [24] it was established that carriers of any of the three founder mutations in *BRCA1/2* were associated with an increased risk in developing high grade prostate cancer. Results showed that *BRCA2* mutation carriers had a 3.2-fold increased risk of high grade prostate cancer. In analysis by Edwards et al. [11] a loss of heterozygosity was evident in the five available prostate cancer tumours from group one which indicated a relationship between *BRCA2* germline mutations and predisposition to prostate cancer in these individuals.

### *BRCA1/2* mutations and prostate cancer survival rate

Within the research it appears evident that a poorer survival rate is greatly associated with *BRCA2* germline mutations. Narod et al. [17] compared the survival of men with a *BRCA1* mutation with prostate cancer with that of men with a *BRCA2* mutation and prostate cancer and found a significant difference in results. The median survival from diagnosis for *BRCA1* was 8.0 years compared to *BRCA2* which was 4.0 years. A similar prevalence was detected in the cohort study by Edwards et al. [11] where the median survival of prostate cancer patients with a *BRCA2* mutation was 4.8 years whereas the median survival in controls was 8.5 years.

Edwards et al. [27] found that five of their six participants carrying *BRCA2* mutations had no family history of prostate cancer and four participants had no family history of breast cancer. This suggests *BRCA2* may be a high risk prostate cancer susceptibility gene. The results also have potential implications for the management of early-onset prostate cancer. It would be reasonable to suggest that such individuals should be offered referral to screening programmes. Utilising this cohort study by Edwards et al. [27], further research was undertaken by the group [11] comparing this cohort with a clinical set from Manchester, UK, of known *BRCA2* mutation carriers with prostate cancer. A small sample of men who possessed a *BRCA2* mutation and were diagnosed with prostate cancer at less than 55 years were compared with men diagnosed at a similar age but without a mutated *BRCA2* gene. Findings concluded that germline mutations in *BRCA2* are an independent prognostic factor for survival in prostate cancer. Such results highlight the importance of developing targeted chemotherapies to treat prostate cancer in men with *BRCA2* mutations [17]. This may be an important factor to consider for future research into treatment.

### *BRCA1/2* mutations in pancreatic cancer

Pancreatic cancer is a disease with poor prognosis and low survival rates worldwide. Its mortality compares strikingly with its incidence. In the UK in 2011, there were 8,773 diagnosed cases and a mortality rate of 8,320 [28]. Through analysis of the literature it was found that both *BRCA1* and *BRCA2* mutations are associated with the incidence of pancreatic cancer and that *BRCA2* mutation poses an increased risk for developing pancreatic cancer [29]. Furthermore environmental and genetic factors have been proposed as causes of the pancreatic cancer with the genetic factor of particular importance believed to be the *BRCA2* gene [30].

It has been reported that pancreatic cancer is the third most common cancer associated with *BRCA1/2* mutations [31]. The risk of pancreatic cancer increases in the individual who has a close relative with the disease. Approximately 5-10 % of pancreatic cancer cases are believed to show familial clustering [32]. As with prostate cancer incidence [27], it has been suggested that patients with pancreatic cancer and germline *BRCA2* mutations tend to be of Ashkenazi Jewish decent and have a younger than average age of onset [33]. Recently, evidence of a strong family history of pancreatic cancer was reported in a study among 211 Ashkenazi Jewish probands. Within the sample, 31 % had a first-degree relative with pancreatic cancer, 53 % had a second-degree relative and 16 % had a third-degree relative diagnosed with the disease; furthermore 26 of the 211 probands had more than one relative diagnosed with pancreatic cancer [29]. It is important to acknowledge that the study participants are of Ashkenazi Jewish decent therefore, it would be reasonable to assume that *BRCA1/2* mutation prevalence may be increased among this sample.

It is clear that *BRCA1/2* mutations are evident in many familial breast-pancreas cancer families and that carriers of the *BRCA2* mutation have an increased risk of developing pancreatic cancer [29]. Nonetheless, the degree to which family history of pancreatic cancer influences the likelihood of detecting a *BRCA1/2* mutation in an individual with breast cancer is less clear [29]. Perhaps, differences in population samples can account for conflicting results within studies making it difficult to make a connection. Furthermore, the use of different analysis models within studies can lead to variations in mutation prevalence.

Pancreatic cancer has been regarded as a component of the breast-ovarian cancer syndrome [29]. In a study by Axilbund et al. [34] over half the study population reported a family history of breast and/or ovarian cancer in addition to pancreatic cancer. The study findings suggest that *BRCA1* mutations are not a substantial cause of breast cancer in familial pancreatic cancer kinships as none of the participants were found to possess a *BRCA1* mutation from DNA sequencing.

Stadler et al. [29] demonstrated that 70 families had more than two relatives diagnosed with breast cancer within the same pedigree as the family history of pancreatic cancer and 31 probands had a relative with ovarian cancer within the same family tree. Axilbund et al. [34] showed that from a sample of 66 pancreatic cancer patients, four reported having had breast cancer prior to being diagnosed with pancreatic cancer. Conversely, in a cohort study by Tulinius et al. [35] no familial risk due to *BRCA2* gene mutations was found for pancreatic cancer among breast cancer patients, yet, it was evident for cancers of the stomach, prostate and kidneys. These results could suggest that specific regions of *BRCA1/2* genes may have increased associations with particular cancers.

Kim et al. [36] reviewed the pedigrees of 1312 families tested for a *BRCA1/2* mutation; 219 families were positive for *BRCA1* mutations and 156 families had *BRCA2* mutations. Results showed that 11 % of the 219 *BRCA1* positive families had at least one relative with pancreatic cancer and 2.7 % had more than one relative with pancreatic cancer. Stadler et al. [29] identified 14.2 % *BRCA1/2* mutations among the sample of 211 Ashkenazi Jews who reported a personal history of breast cancer and a family history of pancreatic cancer. Furthermore, Bermejo and Hemminki [26] demonstrated that families of patients with breast cancer diagnosed before aged 35 years presented significant standard incidence ratios (a ratio that allows comparison of incidence rates among different populations) for pancreatic cancers. However, this standard incidence ratio was reported to be indicative of some association of early-onset breast cancer and pancreatic cancer through causes unrelated to *BRCA1* mutations, although no other proposed causes were suggested.

The findings from the Stadler study [29] demonstrated that approximately 64 % of the relatives with pancreatic cancer were female and 24 % of these women also had a previous diagnosis of breast cancer. Two male relatives, one from a *BRCA1* positive family and one from a *BRCA2* positive family, were diagnosed with both breast and pancreatic cancer. However, the findings from this study suggest that the distribution of *BRCA1* (47 %) and *BRCA2* (53 %) mutations is nearly equal in Ashkenazi Jewish breast-pancreas cancer families who possess a mutation. Conversely, in the study by Axilbund et al. [34], *BRCA1* mutations were not identified despite over half of the pancreatic cancer probands reporting a family history of breast and ovarian cancer. Since the prevalence of *BRCA1/2* gene mutations in the general population is small not all familial clustering of cancers can be associated with mutations in these genes. A strong family history could be attributed to other inheritable factors or lifestyle choices within families.

As with prostate cancer [25], it has been suggested that pancreatic cancer patients who possess a mutated *BRCA2* gene have a younger age of onset [20]. Kim et al. [36] reported the mean age of diagnosis of pancreatic cancer within *BRCA1* positive families was 62.9 years. The median age for males among this group was 59 years and 68 years in females. Within *BRCA2* positive families the mean age at diagnosis was also 62.9 years, while the median age in males was 67 years and 59 years in females. These results support the findings by Stadler et al. [29] where the mean age for diagnosis of pancreatic cancer among the relatives was 67.4 years. This is typically older than the average age at which *BRCA1/2*-related breast and ovarian cancers occur. However, 24 % of the females previously had breast cancer before developing pancreatic cancer.

### *BRCA1/2* mutations in stomach cancer

Stomach cancer represents a significant global cancer burden and *BRCA1/2* mutations have been reported to increase the lifetime risk of developing stomach cancer by as much as 6-fold greater among first-degree relatives of *BRCA1/2* mutation carriers [20, 37]. The risk is reported to be 4-fold greater in *BRCA1* mutation carriers [38] and at least 2-fold greater in *BRCA2* mutation carriers [35].

As with pancreatic cancer [26], familial aggregations of breast cancer and stomach cancer have been highlighted. In a Polish study evaluating the importance of a family history of stomach cancer in predicting the presence of a *BRCA2* mutation in patients with ovarian cancer, findings showed that 8 of 34 women with ovarian cancer and a family history of stomach cancer were found to carry a *BRCA2* mutation versus 3 of 75 women with ovarian cancer and a family history of ovarian cancer but not stomach cancer [39]. In a further study, Jakubowska et al. [40] analysed DNA to determine the frequency and nature of *BRCA2* germline mutations in Polish families where there was a clear aggregation of breast and male stomach cancers occurring at an early age. 29 families with an aggregation of at least one female diagnosed with breast cancer before aged 50 years and one male diagnosed with stomach cancer before aged 55 years participated in the study. They demonstrated that in 12 of 28 families, stomach cancer was diagnosed in a first-degree relative of an early-onset breast cancer proband [40]. This highlights the importance of family history in surveillance.

Tulinius et al. [35] conducted a cohort study using record linkage of breast cancer families to estimate the risk of malignant diseases in families of probands with the same *BRCA2* mutation. Of the 995 probands in the study, 887 were tested for the mutation. 797 tested negative for the *BRCA2* mutation and 90 (10.1 %) tested positive. The relative risk of stomach cancer was significantly increased among the *BRCA2* mutation positive cohort and was reported to be 2.40-fold for first-degree relatives and 1.91-fold for second-degree relatives. Similarly, Bermejo and Hemminki [26] demonstrated that the incidence of individuals affected with stomach cancer before aged 70 years in families with breast and ovarian cancer was 1.88 %, which is significantly higher than in the general population.

Schlebusch et al. [41] demonstrated that stomach cancer prevalence was significantly increased in *BRCA2* mutation families compared to the general population. However, previous to this, van Asperen et al. [42] reported no significant increased risk of developing stomach cancer in their study on cancer risks in Dutch *BRCA2* families. However, Ashkenazi Jews represented 15.5 % of the sample in the Schlebusch study [41] which may have implications for their increased prevalence yet many of their participants were reported to be of Dutch decent. It has been suggested that the frequency of stomach cancer appears greater among Polish families with *BRCA2* mutations [40] but again this could be due to a higher proportion of participants of Jewish decent. The findings from this previous study showed that in the occurrence of breast and stomach cancer among first-degree relatives, *BRCA2* mutations were detected in 16.7 %. Findings from the Jakubowska study [39] suggest the presence of a germline *BRCA2* mutation among these families given the incidence of ovarian and stomach cancer among families in the Polish population. It also confirms stomach cancer is among the range of diseases attributable to *BRCA2* mutations. Figer et al. [43] previously reported the frequency of *BRCA2* mutations among 70 consecutive Ashkenazi Jewish patients with stomach cancer was 5.7 %, approximately five times higher than the general population. Furthermore, Jakubowska et al. [40] showed that *BRCA2* abnormalities were detected in 16.7 % in families where breast and stomach cancers occurred among first-degree relatives and *BRCA2* mutations were identified among 23.5 % in families where stomach cancer occurred among second-degree relatives.

It has previously been suggested that the increased frequency of stomach cancers in *BRCA2* carriers may be sex related as it has been reported to occur primarily in males [20]. In the population based study by Bermejo and Hemminki [26] almost all the individuals diagnosed with stomach cancer in families with breast and ovarian cancers were males. Findings showed that 23 families with breast, ovarian and stomach cancers included 23 men and 1 woman with stomach cancer [26]. It could be argued that as *BRCA1/2* mutations in women can lead to early-onset breast cancer, individuals who develop breast or ovarian cancers early in life may not survive to develop stomach cancer in later life.

The findings from the Polish study by Jakubowska et al. [39] highlight the need for future research to establish if there is a region on the *BRCA2* gene associated with a particularly high risk of stomach cancer. However, a major weakness of these findings is that when constructing pedigrees, cancers in relatives were based on patient recall and pathological confirmation was generally not available. Therefore, it is possible that the risk of stomach cancer in this study may be overestimated or underestimated.

### Impact of *BRCA1/2* mutations in other associated cancers

Previous studies have highlighted an association with *BRCA1/2* gene mutations and an increased risk of developing cancer in sites other than the breast and ovary. The association of *BRCA1*/2 gene mutations with cancer of the prostate, pancreas and stomach has been demonstrated in a number of studies as discussed in this review. However, carcinomas of the colon and kidneys, as well as malignant melanoma, have also been reported to be linked with mutations in the *BRCA1*/2 genes [20, 44]. Nonetheless, the absolute risks for cancer developing at these other sites are small [19]. The strengths of the associations may be difficult to estimate due to the lower reported incidence of these cancers in mutation carriers.

### Screening for *BRCA1/2* mutations

After the discovery of mutations in *BRCA1*/2 genes, widespread interest in genetic testing developed among women at risk of harbouring a disease-associated mutation [45]. Screening and genetic testing has the potential to cause unease within a family as the impact of receiving a positive BRCA mutation diagnosis not only has implications for the carrier, but all family members. Additionally, in some countries, screening for *BRCA1*/2 mutations can result in implications for health and life insurances.

### Implications for male *BRCA1/2* mutation carriers

Kim et al. [36] report that in most *BRCA1/2* testing programmes less than 10 % of the individuals tested are men, yet there is an equal gender distribution in the population of male and female *BRCA1*/2 mutation carriers. It is argued that *BRCA1/2* mutation screening may be of greater relevance to females as the risks of cancer are greatly elevated in female *BRCA1*/2 mutation carriers compared to male [37], however the findings of this review highlight that there are cancer risks for male carriers of *BRCA1/2* mutations therefore screening has relevance for men in their own right, rather than just to inform risk for relatives.

Several motivation factors prompt individuals to undergo genetic screening. In a study by Daly et al. [45] 23 of 26 participants cited concern for their offspring as their main motivation to undergo screening. Concerns about transmitting a mutated gene to daughters appeared to be a major motivating factor. Results reported by Hallowell et al. [46] generated similar findings and found that all men underwent genetic testing with the intention of providing information for their children.

## Conclusions

This review examined freely available, full-text, literature reporting on the impact of both *BRCA1* and *BRCA2* gene mutations and their impact on cancers other than breast or ovarian cancers and identified three main cancers, (other than breast and ovarian,) associated with germline mutations in *BRCA1/2* genes. The majority of the literature proposes an association between *BRCA1/2* mutations and carcinoma of the prostate, pancreas and stomach. Analysis of the selected literature suggests that mutation of the *BRCA2* gene, rather than *BRCA1* gene, has a greater influence on the risk of developing prostate, pancreas and stomach cancers, as well as impacting on the survival and age of onset of these cancers. However, some research has suggested that while there are evident connections between breast and ovarian, and prostate, pancreatic or stomach cancers, it is possible that such links may be due to factors other than possessing a mutated *BRCA1*/2 gene. Other associations may be revealed as further epidemiological information becomes available.

Detailed examination of the literature revealed conflicting findings regarding the association of mutated *BRCA1*/2 genes with familial aggregation of cancers associated with these genes. A number of studies indicated *BRCA1*/2 gene mutations are associated with familial aggregation of cancer. Furthermore, interactions have been established between mutation carrier status of *BRCA1*/2 genes and first-degree family history of cancer. Nonetheless, conflicting findings are evident as other studies demonstrated *BRCA1*/2 mutations were not a major cause of aggregation of cancer in families.

Analysis of the literature identified a number of studies implicating mutated *BRCA1*/2 genes were responsible for a significant fraction of prostate, pancreatic and stomach cancer development, as well as susceptibility to advanced disease. Confirmed associations of *BRCA1*/2 gene mutations with prostate, pancreatic and stomach cancer at a population level have been reported within the research. Nonetheless, some studies have suggested that only a very small proportion of early-onset cancer in the general population is attributed to *BRCA1*/2 gene mutations. It is important to note that because family members share a quantity of their genes and often their environment, it is possible that the large number of cancer cases present in these families may be due in part to other genetic or environmental factors rather than possessing a *BRCA1*/2 gene mutation.

## Abbreviations

BRCA1: Breast cancer susceptibility gene 1
BRCA2: Breast cancer susceptibility gene 2
